# De novo identification of expressed cancer somatic mutations from single-cell RNA sequencing data

**DOI:** 10.1186/s13073-023-01269-1

**Published:** 2023-12-18

**Authors:** Tianyun Zhang, Hanying Jia, Tairan Song, Lin Lv, Doga C. Gulhan, Haishuai Wang, Wei Guo, Ruibin Xi, Hongshan Guo, Ning Shen

**Affiliations:** 1 Department of Hepatobiliary and Pancreatic Surgery of the First Affiliated Hospital & Liangzhu Laboratory, Zhejiang University School of Medicine, Hangzhou, 311121 China; 2 Kidney Disease Center, the First Affiliated Hospital, Zhejiang University School of Medicine, Zhejiang, 311121 China; 3 Department of Biomedical Informatics, Harvard Medical School, Boston, MA 02115 USA; 4 College of Computer Science, Zhejiang University, Hangzhou, 311121 Zhejiang China; 5 Zhejiang University-University of Edinburgh Institute, School of Medicine, Zhejiang University, Jiaxing, 314400 China; 6 School of Mathematical Sciences and Center for Statistical Science, Peking University, 5 Yiheyuan Road, Beijing, 100871 China; 7 Bone Marrow Transplantation Center, The First Affiliated Hospital, Zhejiang University School of Medicine, Hangzhou, 310003 Zhejiang China

**Keywords:** Somatic mutations, Single-cell RNA sequencing data, Recurrently Expressed SNV Analysis, High precision

## Abstract

Identifying expressed somatic mutations from single-cell RNA sequencing data de novo is challenging but highly valuable. We propose RESA – Recurrently Expressed SNV Analysis, a computational framework to identify expressed somatic mutations from scRNA-seq data. RESA achieves an average precision of 0.77 on three in silico spike-in datasets. In extensive benchmarking against existing methods using 19 datasets, RESA consistently outperforms them. Furthermore, we applied RESA to analyze intratumor mutational heterogeneity in a melanoma drug resistance dataset. By enabling high precision detection of expressed somatic mutations, RESA substantially enhances the reliability of mutational analysis in scRNA-seq. RESA is available at https://github.com/ShenLab-Genomics/RESA.

**Supplementary Information:**

The online version contains supplementary material available at 10.1186/s13073-023-01269-1.

## Background

Somatic mutations are accumulated during cell generations and can transform normal cells into cancer, promote tumor progression, and develop drug resistance. Genetic heterogeneity together with transcriptional heterogeneity are two key aspects that contribute to cancer evolution and drug resistance. Recent studies have attempted to identify somatic mutations, single nucleotide variants (SNVs) in particular, by associating them with transcriptional variations in cancer bulk RNA-seq data [1, 2]. However, it remains challenging to connect transcriptional heterogeneity to genetic heterogeneity at the single-cell level. Therefore, identifying somatic mutations carried by RNA at the single-cell level is highly valuable.

Experimental technologies, such as targeted genotyping coupled with scRNA-seq, have been developed to enable the study of expressional and somatic variations together at single-cell level [3–5]. However, the single cells have limited amounts of biological materials, a small number of detectable mutations, and a low signal-to-noise ratio have restricted their application. As a result, computational methods are sought-after to identify expressed somatic mutations from scRNA-seq data. Profiling of scRNA-seq coupled with bulk genotyping, e.g. whole exome sequencing (WES), whole genome sequencing (WGS), and single cell genotyping of selected mutations, of the same sample have been applied to study intratumor heterogeneity and lineage tracing [5–10]. Although such data can effectively minimize false positives, their design requires thoughtful consideration and can only characterize a limited number of mutations, thus they were not widely adopted.

Computational methods that identify expressed somatic mutations directly from scRNA-seq data de novo are highly desirable, as such methods not only provide orthogonal insights into the intratumor heterogeneity but also face fewer experimental challenges. Most mutational analysis of scRNA-seq use data generated from scRNA-seq technologies with full length library prep, e.g. SMART-seq2 [11], for its higher coverage in the gene body compared to the 10X genomics and Drop-seq approach. De novo somatic mutation calling and follow-up analysis have been applied to various biological questions including aging in the human pancreas, Alzheimer’s disease, glioblastoma, lung cancer, etc. [12–15]. Methods reported in these studies employ standard variant calling steps that involve quality-based filtering such as sequencing quality or stratification from a curated whitelist of known cancer mutations, following the standard variant calling steps. However, these methods were originally designed for bulk RNA-seq data and their reliability for single-cell RNA-seq data still requires further evaluation [16–19].

In this study, we developed a computational framework named Recurrently Expressed SNV Analysis (RESA) that can detect expressed somatic SNVs with high precision directly from scRNA-seq data. RESA is composed of a specific filtering workflow tailored for scRNA-seq data, especially the recurrence of expressed SNVs across cells. Additionally, we introduced a joint logistic regression (RESA-jLR) model that expands the pool of somatic variants by leveraging information from earlier steps. To evaluate the performance of RESA and RESA-jLR, we conducted in silico spike-in experiments using over 800 cells from the human pancreas. Furthermore, we benchmarked RESA and RESA-jLR using real scRNA-seq datasets with matched WES data from 15 cancer cell lines and 4 tumor PDX tissues. RESA is specifically designed for high precision detection of somatic mutations while minimizing noise and artefacts from experimental procedures, so that users can be confident with the reliability of the results (Additional file 1: Fig. S1). Thus, the applications of RESA and RESA-jLR may provide a reliable and integrative view to study intratumor heterogeneity and drug resistance.

## Methods

### WES and scRNA-seq datasets of cancer cell lines

The exonic somatic mutations for all cancer cell lines tested in this study were processed by the CCLE project and downloaded from CCLE_20Q1_mutations [20]. The scRNA-seq for cancer cell lines were downloaded from GSE105451, GSE76312, GSE99795, GSE150993, GSE108383, GSE140440, and GSE69405 respectively.

Specifically, scRNA-seq for JURKAT, SET2 (GSE105451), and K562 (GSE76312) was done using the SMART-seq + and TARGET-seq technologies [4, 5]. All other cell lines were done with the Smart-seq2 protocol. LNCaP (GSE99795) was treated with double thymidine for 12 h (hr) to synchronize the population cell cycle, then single cells were collected at 0 h without drug treatment, and 12 h with and without drug treatment [21]. HCT116 (GSE150993) live cells and methanol-fixed cells were processed and sequenced respectively [22]. Yu-jui Ho et al. sequenced A375 (GSE108383) with and without BRAF inhibitor [23]. Similarly, Patricia M Schnepp et al. divided their cells into docetaxel-sensitive and -resistant groups of DU145 (GSE140440) [24]. Finally, we analyzed both the cancer cell line NCI-H358 data (GSE69405) and lung adenocarcinoma (LUAD) PDX tumor data from Kyu-Tae Kim et al. [25].

Besides, we evaluated the consistency between two experimental replicates of these cell lines after applying RESA-jLR in all applicable cases. We counted the number of detected mutations in each replicate and computed the fold change between the number of detected mutations in replicate 1 and the number of detected mutations in replicate 2. If the result is close to 1, it means the number of detected mutations between two replicates is similar. The consistency shows that our method has stable performance in datasets under similar conditions.

### RESA workflow

**Fig. 1:**
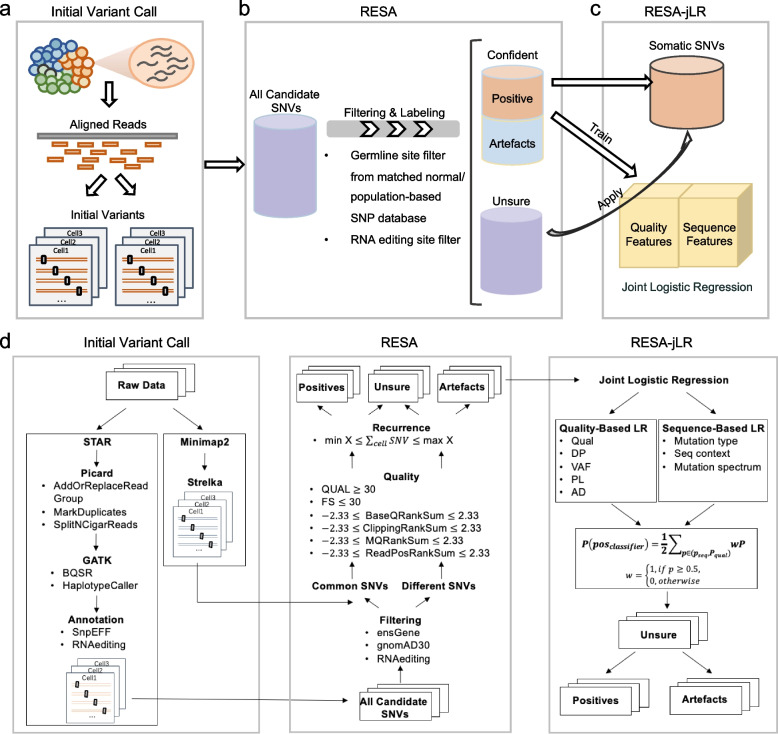
RESA workflow. **a** Step1 is an initial variant call using two aligners and two mutation calling algorithms. **b** RESA: Variants calling then goes through a series of filtering and labeling, categorizing into a confident set of positive somatic variants and artefacts, and a set of unsure SNVs to refine. **c** RESA-jLR: The confident set of variants is used to build a joint logistic regression model, where the model is applied to make predictions in the unsure set of SNVs to refine and expand the final positive set of somatic SNVs. **d** the detailed workflow of RESA

RESA comprises three main steps: initial variant calling, filtering and labeling, and modeling and refinement [26]. The first step of RESA is to align the sequencing reads to the genome and run through basic mutation calling algorithms with RNA-seq specific parameter settings (Fig. 1a). To minimize aligner and variant caller biases, two independent alignments and two basic variant callers were employed. Next, we run through a series of processing, filtering, and most importantly, cross-cell recurrence counting. In this step, the variants are grouped into 3 categories: 1) a high-confidence set of SNVs that are either putative somatic SNVs or artefacts; 2) a set of filtered-out variants; and 3) a set of undefined SNVs for further refinement (Fig. 1b). The high-confidence set of putative positive somatic variants were labeled as RESA-identified somatic mutations.

Denoting RESA identified mutations as “positive” and the putative biases and artefacts variants as “negative”, RESA randomly splits the data into training and test sets. We developed a joint logistic regression classifier that models both quality and sequence-related features independently (Fig. 1c). The joint logistic regression classifier was then applied to make predictions on the set of undefined SNVs. The final set of somatic SNVs identified by RESA-jLR is a combination of positive SNVs from the high-confidence set and predicted positive SNVs from the undefined set using the joint logistic regression model.

### Initial read mapping and variant calling

To reduce the bias in aligners and variant callers, we employed two independent alignment and variant calling methods for each cell's fastq file in the scRNA-seq analysis. The first method consisted of aligning the reads using the widely adopted STAR aligner [27], a highly effective read aligner, in a two-pass mode to improve alignment accuracy. Then processing the aligned reads based on recommendations from GATK (gatk/4.0.0.0) [28] involving steps such as removing duplicates and recalibrating base quality scores and calling variants using GATK (Fig. 1d). The second method involved aligning the reads using the Minimap2 aligner [29], followed by RNA variant calling settings from Strelka [30] (Fig. 1d). Only variants marked as passing the default filtering set by the Strelka algorithm were retained for this method. Therefore, we obtained two sets of candidate variants per cell that were detected by these two pipelines. Our primary reference for downstream analysis was the STAR-GATK procedure which is a widely used combination of read alignment and variant calling pipelines, as we found that the GATK process can aid in identifying and correcting noise and artefacts in the data. The outputs from the STAR-GATK procedure included information that was utilized in subsequent steps.

### RESA

We then annotated and filtered variants from the STAR-GATK pipeline similar to the CTAT-Mutations Pipeline which is designed to detect variants from bulk RNA sequencing data [31]. We first annotated the variant location using ANNOVAR and kept only SNVs located in the exonic region for downstream filtering and labeling. Then SNVs that overlapped with RNA editing databases were removed for potential RNA editing events (Fig. 1d). After that, we filtered germline variants generated from matched normal if available, or from population SNP databases such as the genome Aggregation Database(gnomAD) 3.0. Hence, the primary filtered candidate variants from the original STAR-GATK pipeline were kept for further procession.

Next, we checked the concordance between the filtered STAR-GATK pipeline and the second pipeline using RESA. Variants identified by both pipelines were considered for candidate variant set A which represented the intersection of results from these two pipelines, while the variants of the filtered STAR-GATK pipeline which were the elements of the second pipeline were considered for candidate set B. RESA further processed the variant calling outputs by setting additional requirements on read quality, variant quality, strand bias, and variant positional bias. The common variants from the above-mentioned procedures were required to have a minimum read depth of 3 or user-specified site-specific depth.

Importantly, the resulting filtered SNVs for variant set A were stricter or at least comparable to quality-based filtering approaches used in other studies, such as the Quality-filter approach [12, 16] referred to as Enge, 2017, for performance comparison.

RESA assumes that cancer cells evolve in a clonal manner and thus expressed somatic mutations have cross-cell recurrence, whereas the noise and artefacts are likely distributed randomly with a small probability of recurrence across the cell population (Fig. 1d). Based on this assumption, RESA set a cross-cell recurrence filter on a pseudo-bulk basis by keeping only SNVs that were detected in at least X number of cells to filter out artefacts, where X (X >  = 3) by default is no less than 10% of the total cell number and can be adjusted by users (Fig. 1d), which removed background sequencing errors and artefacts from candidate somatic SNVs. This criterion was intended to filter the false positive somatic mutations because we assume that the true positive somatic mutations should at least express a certain number of cells, but it may also remove true positive somatic mutations that are only expressed in very few cells (less than 10%). Furthermore, mutations detected in more than 80% of the total cell number were considered to be germline polymorphisms or artefacts as well. The key assumption is that compared with somatic mutations, germline variants should be detected in most cells at the RNA level.

The upper and lower limit number of cross-cell recurrence were used as parameters in the software and could be adjusted in a user-specified manner. The filtered SNVs from set A were considered putative true somatic variants. Thus, RESA built a putative “true positive” set of somatic variants directly from scRNA-seq data based on cross-cell recurrence and quality-based filtering.

RESA applied several criteria to enable filtering of putative germline mutations: 1) germline variants that were generated from the matched normal data, if available and specified in user input; 2) putative germline variants identified from population SNP databases such as gnomAD 3.0.; 3) putative germline variants with high detection recurrence in the tested cell population.

To build a high-confidence set of noise and artefact SNVs, RESA selected SNVs from set B that failed the above-mentioned criteria, such that putative noise and artefacts had the following properties:1) SNVs did not pass the quality-based filtering as described above, and 2) SNVs did not show any cross-cell recurrence. The remaining SNVs in set B pass the above-mentioned criteria and SNVs in set A failed the criteria were defined as the unsure set, which was subjected to RESA-jLR described below. We applied this selection criteria for each single cell and found very few SNVs overlapping between the positive and negative sets after merging SNVs across the cell population, suggesting a decent separation between our putative positive and negative sets.

### RESA-jLR

RESA employs a process of combining all putative positive and negative sets of somatic SNVs. The input variants to feed RESA-jLR combined all variants in positive and negative sets. Then the input dataset was split into training and test sets with the 3:1 ratio, with 3 quarters of the data used for training the model, and a quarter of the data used as an independent test set to evaluate the model performance. Because the sample size for each class on the training set was often imbalanced, thus, random oversampling was applied to replicate observations in minority classes, thereby rebalancing the dataset. RESA with the joint logistic regression model was the joint composition of two logistic regression models. One model depended on quality-based features such as variant quality, read depth, variant allele fraction, normalized probabilities of genotype, and allele depth. The other model derived its features from sequence-based attributes like mutation types, sequence contexts, and mutation signature components. Quality-based features were generally weighted similarly across datasets, while mutation sequence composition can be more sample-specific; hence, they were modeled differently. We trained the joint logistic regression model using the liblinear library, with L1 regularization applied to the quality-based model and L2 regularization applied to the sequence-based model using one-hot encoding. Each logistic regression model returned probability values for positive and negative classifications, with users being able to specify their thresholds based on these probabilities. Then we combined these two models into an integrated classifier with the following equation:

$$P\left({pos}_{classifier}\right)=\frac{1}{2}{\sum }_{P\in ({P}_{seq}, { P}_{pos})}wP,$$

Where $$w=\left\{\begin{array}{c}1, if \,P \ge 0.5\\ 0, otherwise\end{array}\right.$$, ${P}_{seq}$ and ${P}_{pos}$ were probabilities to be the positive class of the two regression models. RESA-jLR sets 0.5 as the default threshold, which meant RESA-jLR defined the SNVs as positive if $P\left({pos}_{classifier}\right) \ge 0.5$. We also included probability as a parameter so that users could modify the thresholds.

After training, we assess the model's performance on the test set using the AUC score. As opposed to accuracy, the AUC score is appropriate for imbalanced datasets. Additionally, we applied the model to the candidate SNVs in the unsure set to refine and extrapolate the putative somatic variant set. This step recovered some true somatic variants filtered out in stringent criteria described earlier and enhanced sensitivity while maintaining high precision.

### Somatic mutation detection by other methods

We conducted a performance evaluation of RESA and RESA-jLR by comparing them to five previously published methods, including Enge 2017, Maynard 2020, BCFtools, VarScan, and Hovestadt 2019. We applied similar filtering criteria and parameters for somatic mutation filtering across all methods.

Somatic mutations were filtered by similar quality criteria which have been adopted in Enge 2017 [12, 16]. Specifically, we aligned sequencing data to the hg38 human reference using STAR and called alignment BAM files for different cell lines using GATK HaplotypeCaller. We then filtered out potential artefacts using VariantFilter (-cluster 3; -window 35; -filter QD < 2.0; -filter FS > 30.0). Finally, we employed the GATK variant quality score recalibration pipeline to filter the variation calls and exclude known germline variants.

Moreover, we utilized concord whitelist-based mutation selection which has been adopted in the Maynard 2020 with similar filtering criteria [15]. Reads were aligned using STAR aligner, processed as suggested by GATK pipeline (gatk 4.0.0.0). Variants were called by GATK HaplotypeCaller (this process is adopted as part of the STAR-GATK Pipeline) [30]. Next, we filtered out variants using the following criteria: 1) variants are located in exonic region; 2) variants have pathogenic effect as predicted by FATHMM; 3) variants have minimum total read depth of 3; 4) variants have been curated in COSMIC database [32] in the same tissue type as sample tested. Besides, we also applied the STAR-GATK Pipeline [28] from raw sequencing data detecting variants.

BCFtools [33] (BCFtools 1.9) and VarScan (Varscan v2.4.4) [34] were evaluated for their performances of detecting mutations across scRNA-seq data [16]. We aligned reads to hg38 human reference using the BWA-MEM (BWA 0.7.17) and then used the GATK-PICARD pipeline to preprocess the BAM file. For better comparison, we chose the default parameters of these toolkits.

Besides, we created another recurrence-based method based on recurrence rates by following filtering criteria in the Hovestadt 2019 [10]. We quantified variants at the genomic position using SAMtools mpileup for preprocessed BAM files in scRNA-seq data and then filtered out variants detected in fewer than three cells or not detected in the genome sequencing data.

### Somatic mutation detection using RESA in in silico spiked-in dataset

In order to further assess the effectiveness of RESA on human tissue datasets, we conducted a test using in silico spiked-in datasets. We hypothesized that scRNA-seq data from healthy juveniles would not contain somatic mutations, with the variants detected more likely to be germline mutations or artefacts resulting from sequencing. To assess the ability to detect somatic mutations in human tissue RNA datasets, we added somatic mutations from 10 distinct cell lines. The inclusion of various types of somatic mutations from different cell lines allowed for the evaluation of the generalization of RESA, as it was exposed to diverse mutation spectrums. And in this experiment, our focus was on the ability to detect add-on somatic mutations to avoid the potential impact on model performance that could arise from using ground truth data filtered by different criteria.

To avoid the subjective factors and controllable preferences, we used an independent tool – BAMSurgeon, a tool that can introduce somatic mutations to BAM files, to generate simulation data [35]. To add mutations, we collected three independent real scRNA-seq data of pancreatic epithelial cells from healthy juveniles with SMART-seq2 technology, aged 1 month, 5 years, and 6 years [12]. We aligned the raw reads from real scRNA-seq data to GRCh38 (GRCh38.p12) using BWA-MEM 0.7.17 with default parameters to create the original BAM files.

To spike SNV mutations into each scRNA-seq dataset, we used ten cancer cell lines with varying numbers of mutations from CCLE [20] as the 'truth' VCF files that contained variant position information and variant allele fraction (VAF). The VAF of SNVs was determined by WES. If the VAF was missing, we replaced the VAF with 0.5. We used addsnv.py with the following relevant settings to spike SNVs in the original BAM files of cells: –mindepth 2 –minmutreads 1 –aligner mem. We then converted the 'burned-in' BAMs to in silico 'spiked-in' FASTQ files using GATK 4.2.3.0 SamToFastq. We identified 'spike-in' SNVs from each cell and reintroduced simulated SNVs to 50% of VCF files generated by the STAR-GATK procedure. Once algorithmic biases were introduced into the cells, we proceeded to analyze the 'spike-in' data using RESA and five other algorithms—Enge 2017 [12], Maynard 2020 [15], Hovestadt 2019 [10], BCFtools [16], and VarScan [16].

### Method evaluation

To evaluate the performance of different methods, we gathered somatic mutations identified by various methods as mentioned above. We defined WES-identified somatic mutations that were carried in the tested scRNA-seq dataset as the ground truth. In this context, false positives are "false" somatic mutations including germline variants, RNA editing sites, noise, and artefact mutations in scRNA-seq data that are labeled as somatic mutations by RESA. Meanwhile, false negatives refer to true somatic mutations in scRNA-seq that are mislabeled by RESA as "false".

Precision, sensitivity, and F0.5 score were utilized to assess the performance of RESA across datasets. Precision evaluates the proportion of true positives among all the predicted positives, while sensitivity measures the proportion of true positives among all the ground truth positives. The F0.5 score is a commonly used adjusted F-score that assigns greater weight to precision than to sensitivity, given that precision is of greater importance in our algorithm. The F0.5 score satisfies the following equation:

$$F_{0\cdot5}=1.25\cdot\frac{\left(precision\,sensitivity\right)}{0.25\,precision+sensitivity}$$

As we developed RESA, our strategy prioritized precision over sensitivity to instill greater confidence. This means our focus was on detecting true somatic mutations while minimizing the impact of artefacts. Even in situations where there is no matched normal WES/WGS data available for comparison, our strategy could still accurately predict somatic mutations.

To show the consistency between replicate experiments, we have presented it by using the overlap coefficient. It is defined as the size of the intersection divided by the smaller of the size in these two datasets.

$$overlap \left(X,Y\right)=\frac{\left|X\cap Y\right|}{{\text{min}}\left(\left|X\right|,\left|Y\right|\right)}$$

### Analyzing primary tumor data

Kyu-Tae Kim used scRNA-seq by SMART-seq and whole-exome sequencing (WES) to examine intratumor heterogeneity in lung adenocarcinoma tumors (GSE69405). A 60-year-old male patient had a treatment-naïve lung cancer tumor, which was surgically excised and inoculated into immunocompromised mice to generate patient-derived xenograft (PDX) tumors. A sample of PDX tumor containing 34 single cells and an additional sample with 43 cells as a biological replicate were sequenced. WES analysis was also done on the patient's blood to obtain the matched normal data and filter out germline mutations.

The raw reads for melanoma scRNA-seq used for mutation identification were obtained from GSE116237 [36]. The matched WES data for the PDX sample with two replicates were downloaded from EGAD0000179 [37]. Four time points (T0, phase 1, phase 2, and phase 3) were included in the scRNA-seq dataset, but only T0 and phase 3 had matched WES data. To assess the performance of our method using matched WES data, we utilized RESA and RESA-jLR separately on these two time points.

Somatic mutation calling from WES data was carried out similarly to the CCLE project, including aligning WES reads to the GRACh38 reference genome using BWA-MEM 0.7.17, marking duplication with Picard, and avoiding systematic errors by Base Quality Score Recalibration with variant sites identified from the 1000 Genomes Project, dbSNP138, and Mills and 1000G gold standard indels. HaplotyCaller was used to call SNVs for the PDX tumor sample that contained matched normal data such as patient blood WES. The model of scoring variant quality for filtering was developed by VariantRecalibrator and ApplyVQSR under default parameters.

### Re-analyzing melanoma scRNA-seq expression matrix

The gene expression read count table after processing melanoma PDX data for all cells was obtained from GSE116237. The Seurat package was employed for expression analysis, and read counts were log normalized. The top 2000 variably-expressed genes were selected for dimension reduction using PCA. The K-nearest neighbor (KNN) graph was clustered and refined using the Louvain algorithm based on the top 30 transformed PCs from PCA. UMAP was used with a dimension of 20 and applied to the top 30 PCs for data exploration and visualization.

### Analyzing stage-specific somatic mutations

To enhance the sensitivity of our methods, we utilized RESA and RESA-jLR on the melanoma scRNA dataset across four time points. We then assessed stage-specific enrichment by calculating the *p*-value based on the cumulative distribution function of the hypergeometric distribution. We aimed to determine whether the number of cells with a mutant gene was overrepresented in one stage compared to the other stages. For each candidate gene containing a somatic mutation, we counted the number of mutation-carrying cells in each tumor stage and computed the p-value to identify the enrichment at each stage. If a gene passed the defined threshold p-value of 0.05 at a specific stage, we labeled it as enriched at that stage. This indicates that the distribution of the number of mutation-carrying cells is non-uniform and significantly enriched at the specific stage compared to the other stages [38].

### Custom analysis and plots

Mutation Signature plots were done using SigProfilerPloting. UMAP plots were done using the Seurat package in R. Other custom plots were done using the Seaborn package in Python and the ggplot2 package in R.

## Results

### Challenges of detecting expressed SNVs in scRNA-seq data

**Fig. 2:**
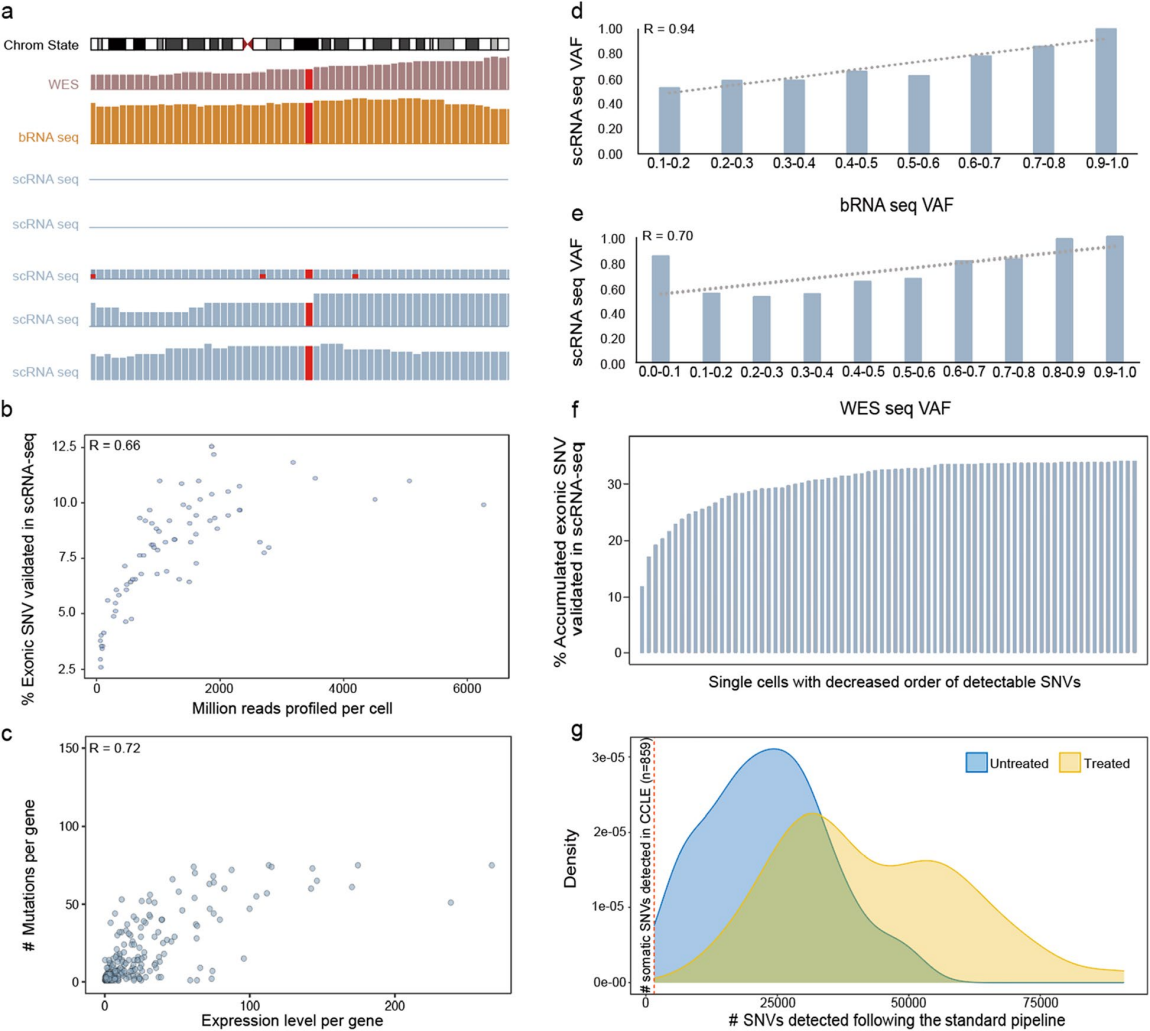
Detecting expressed somatic mutations in the A375 cell line and pancreas tissue datasets. **a** Integrative Genomics Viewer (IGV) window shows the hotspot mutation *BRAF* V600E in the A375 cell line. **b** The scatter plot of the percentage of exonic SNVs validated in scRNA-seq against the million reads per cell. **c** The scatter plot shows the expression level against the number of expressed somatic mutations in each gene. **d**, **e**, The bar plot illustrates the distribution of VAF of expressed somatic mutations in scRNA-seq compared with corresponding VAF in bRNA-seq (**d**) and in WES (**e**). **f** The percentage of accumulated expressed SNVs validated in scRNA-seq. **g** The density plot shows the distribution of detected SNVs of each cell after passing the standard variant calling pipeline in the A375 cell line datasets with two conditions

De novo identification of somatic SNVs from scRNA-seq data is challenging due to the sparsity and noisiness of the data. Several factors, including allelic expression, expression abundance of mutant-containing genes, sequencing coverage per cell, variant allele frequency (VAF), and clonality, may contribute to the sparsity of the detectable mutations. To understand various factors that may influence the detectability of somatic mutations in scRNA-seq data, we gathered WES, bulk RNA-seq (bRNA-seq), and scRNA-seq datasets for the melanoma cell line A375. For example, the site for the *BRAF* V600E driver mutation has high coverage (referred to as site-specific depth hereafter) in both WES and bRNA-seq data, whereas only three out of five selected single cells have detectable site-specific depth (Fig. 2a). As indicated by this example, allelic expression as well as sequencing coverage might contribute to the sparsity of somatic mutations detectable from scRNA-seq data, which is also known as the effect of “allelic dropout”. Next, we analyzed scRNA-seq of all A375 cells and observed a significant correlation between sequencing coverage and expressed somatic mutations per cell (Fig. 2b). Concordantly, both site-specific depth and the gene expression levels correlate positively with the number of detectable somatic mutations (Fig. 2c, Additional file 1: Fig. S2). In addition, scRNA-seq VAF correlates positively with VAF from both bRNA-seq (Fig. 2d) and WES (Fig. 2e, Additional file 1: Fig. S3). Collectively, these results suggest that the detection of somatic mutations in scRNA-seq is jointly shaped by the mutation burden, sequencing coverage of the cell, as well as the sequencing depth and expression level of the mutant allele.

The sparsity of detectable somatic mutations in scRNA-seq data can be alleviated by sequencing a large number of cells (Fig. 2f). However, de novo identification of expressed somatic mutations following standard variant calling pipelines results in tremendous amounts of noise in the data (Fig. 2g). One possible explanation for the noise is that currently used mutation calling algorithms failed to take into account the noise and artefact SNVs generated during the experimental procedure. Consistently, a recent study evaluated the performance of several mutations calling algorithms on scRNA-seq data using simulated scRNA-seq reads, and demonstrated that most of the algorithms tested showed satisfying performance with FDR < 0.05 [16], suggesting that noise and artefacts carried by the sequencing read can be picked up equally well with actual mutations of the cell. This aligns with our knowledge that the experimental procedures of scRNA-seq, including cell lysis, cDNA conversion, library preparation, etc., may result in arteficial variants that far exceed the number of true variants (Additional file 1: Fig. S4). Subsequently, the direct application of such mutation calling algorithms on real scRNA-seq data may mislead biological interpretations of the data. Therefore, computational methods that can effectively filter out such experimental noise and artefacts are essential for de novo identification of somatic mutations in scRNA-seq data.

### The computational principle of RESA to identify somatic SNVs from scRNA-seq

We present a computational framework RESA – Recurrently Expressed SNV Analysis, which detects expressed somatic mutations with high precision directly from scRNA-seq data (Fig. 1a-d). We focused on scRNA-seq technologies that capture full length transcripts and single nucleotide variants (SNVs) for somatic mutation analysis. RESA effectively eliminates noise and artefact variants in the scRNA-seq data by applying a series of filtering steps, including cross-cell recurrence. For instance, with the standard variant calling process, about 0.5–0.8 million SNVs were identified in cancer cell lines A375 and HCT116, where numerous noises and artefacts dominate the pool (Additional file 1: Fig. S5). We found that different variant callers followed with basic filtering barely helped. On the contrary, applying RESA effectively removed the majority of noises and artefacts, and achieved about 75% of true somatic SNVs out of the total SNVs identified by the algorithm (Additional file 1: Fig. S5c, d). In addition, we developed a joint logistic regression approach following RESA, i.e., RESA-jLR, to filter out noise and artefacts while increasing sensitivity by identifying positive cases from an unsure set of variants. Notably, applying RESA-jLR identified additional somatic SNVs while keeping the proportion of noise and artefacts small (Additional file 1: Fig. S5).

We validated that RESA-identified somatic mutations maintained a positive VAF correlation between scRNA-seq and WES, as well as scRNA-seq and bRNA-seq across multiple cell lines (Additional file 1: Fig. S6a, b). In particular, although germline variants are thought to share similar quality properties as somatic mutations, we adopted several approaches to help eliminate germline variants in different circumstances.

We applied RESA-jLR to full-length scRNA-seq data from 15 datasets encompassing 8 cancer cell lines, which were generated by different research groups, employed different experimental protocols, and operated under different experimental conditions (Methods, Additional file 2: Table S1). To assess the accuracy of the joint logistic regression model, we evaluated its performance in the independently held-out test sets. The model achieved an AUC ranging from 0.79 to 0.98, with an average AUC of 0.89 (Additional file 1: Fig. S7), suggesting the joint logistic regression model was highly effective. Furthermore, we observed high agreement among most datasets when comparing the feature weights of quality-related features in the quality-based logistic regression. This indicates that the effect of quality-related features on mutation detection could be generalizable (Additional file 1: Fig. S8a). In contrast, we found that feature weights of sequence-related features were cell type-specific, with high correlation observed only among different experimental conditions of the same cell line (Additional file 1: Fig. S8b). This demonstrated the adaptable nature of RESA-jLR to specific cell types and the robustness of RESA-jLR across experimental conditions.

### RESA maintains high precision in in silico spike-in scRNA-seq datasets of human tissue

**Fig. 3:**
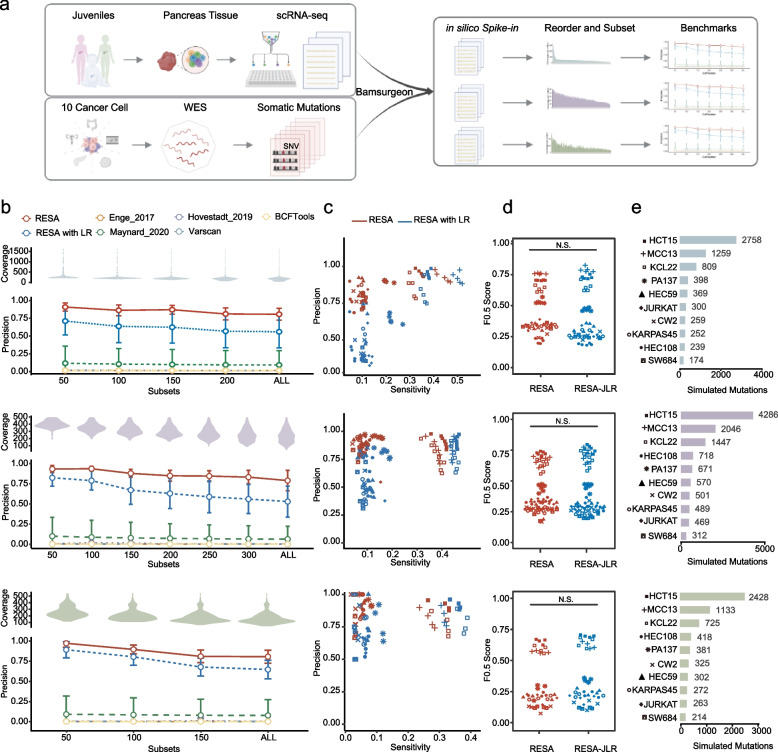
Benchmark RESA to other methods in the in silico spike-in scRNA-seq datasets. **a** Workflow for in silico spike-in. scRNA-seq raw reads of pancreas tissues from 3 healthy juveniles using SMART-seq2 technology in Enge et al. 2017 [12] were collected as original BAM files. Somatic SNVs of 10 cancer cell lines covering 5 tissues of origin were identified from WES data. Bamsurgeon spiked cancer cell line somatic SNVs into the original BAM files to produce ‘Burn-in’ BAM files. In silico spike-in scRNA-seq datasets were ordered by the coverage of each cell, split into several subsets, and followed by further evaluations. b, The violin plot illustrates the distribution of coverage in each subset. The error bars display the average and standard deviation of the precision of RESA, RESA-jLR, and the other 5 previously published algorithms. If the minimum value of the error bar is less than 0, 0 is shown. Top: a 4-month-old infant (Blue). Middle: a 5-year-old child (Purple). Bottom: a 6-year-old child (Green). **c** The scatter plot shows precisions and sensitivities in different subsets of 10 cell lines. Points in red are the results of RESA, and points in blue are the results of RESA-jLR. Top: a 4-month-old infant. Middle: 5-year-old child. Bottom: a 6-year-old child. **d** F0.5 score in in silico spike-in scRNA-seq datasets (Wilcoxon rank-sum test, NS: not significant, * *p* < 0.05, ** p < 0.01, *** *p* < 0.001, **** *p* < 0.0001). Top: a 4-month-old infant (Blue). Middle: 5-year-old child (Purple). Bottom: a 6-year-old child (Green). **e** The bar plot illustrates the number of “spiked-in” mutations across 10 cell lines. Top: a 4-month-old infant (Blue). Middle: 5-year-old child (Purple). Bottom: a 6-year-old child (Green)

To comprehensively benchmark RESA against other methods in human tissue scRNA-seq data under diverse scenarios, we designed an in silico spike-in experiment (Fig. 3a). Briefly, we collected SMART-seq2 data generated from pancreas tissues of three donors as three baseline datasets, namely 4-month-old (221 cells), 5-year-old (331 cells), and 6-year-old (178 cells), respectively [12]. We considered the datasets from these donors to carry predominantly noise and artefacts and expected minimal true somatic mutations as they were generated in body cells after birth. We then collected a total of 77,088 somatic mutations from CCLE WES data of 10 cancer cell lines with varying cancer types and mutation burdens. For each cancer cell line and each baseline dataset, we “spiked-in” somatic SNVs in silico using BAMSurgeon [35], followed by performing RESA or other methods to detect somatic mutations. To analyze the effect of coverage per cell and cell number on algorithm performance, we sorted the single cells in decreasing coverage and tested algorithms’ performance on different subsets of the cell population (Fig. 3a, Methods).

RESA demonstrated the most consistent and highest precision in all spiked-in scRNA-seq datasets tested, with average precision of 0.84, 0.87, and 0.87 for the three baseline datasets, respectively, followed by RESA-jLR model with average precision of 0.61, 0.66, and 0.76, respectively (Fig. 3b). Among the five previously published methods, Maynard et al. showed the highest average precision. However, the precision varied significantly across different test cases, with an average precision of only 0.2 for all three baseline datasets (Fig. 3b). Furthermore, spike-in results showed that additional cells with lower sequencing coverage led to decreased precision overall, but RESA demonstrated more stable precision compared to the RESA-jLR model (Fig. 3b). This suggests that sequencing coverage at a single cell level might be more crucial for mutation detection than the total number of cells tested. Additionally, we observed that RESA consistently maintained high precision with low variation even with a significant variation in the number of “spiked-in” somatic mutations from different cancer cell lines (Fig. 3b, d). Here we demonstrated that RESA can reach average an precision of 0.86 in primary tissue samples, with an average of 1.8 million reads/cell (Additional file 1: Fig. S9). Notably, in the in silico spike-in data of a 1-month-old infant, more than 50 cells had less than 1 million reads, yet the average precision for this dataset was approximately 0.87.

The RESA-jLR model complemented the conservative approach used in RESA by demonstrating increased sensitivity at the cost of decreased precision. For instance, the RESA-jLR identified, on average, 20% more new somatic mutations in the test cases from the 5-year-old baseline dataset (Additional file 1: Fig. S10). We further evaluated the performance using an F0.5 score, a weighted harmonic mean emphasizing more on precision over sensitivity. In test cases with large numbers of “spiked-in” somatic mutations, RESA-jLR outperformed RESA with an increase in sensitivity and a stable precision that resulted in higher F0.5 scores (Fig. 3c, d, e, Additional file 1: Fig. S11), suggesting that RESA-jLR has advantages in datasets with high mutation burden. In addition, through in silico “spike-in” analysis, we demonstrated the superior performance of RESA across different sequencing coverage and cancer cell lines. Our results highlighted the difficulty in balancing precision and sensitivity in scRNA-seq somatic mutation detection tasks.

### RESA achieves higher precision across cancer cell line data

**Fig. 4:**
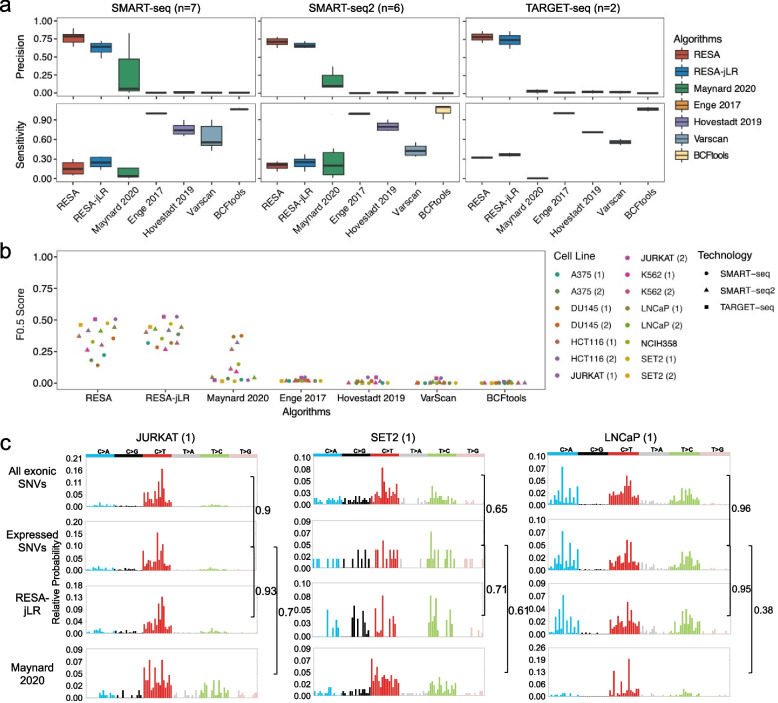
Evaluating the performance of RESA with comparison to other methods using WES data across multiple cancer cell lines. **a** Boxplots showing precisions (top) and sensitivities (bottom) of different methods in identifying positive somatic SNVs using WES data as ground truth across 15 scRNA-seq datasets. **b** The scatter plot showing F0.5 scores of different methods in identifying positive somatic SNVs using the number of somatic SNVs in WES data as ground truth across 15 scRNA-seq datasets. **c** Mutation spectra of somatic SNVs identified using all exonic SNVs, all expressed SNVs, RESA-jLR, and the Maynard 2020 approach across 3 scRNA-seq datasets. Pairwise cosine similarity scores were shown next to brackets

The WES/WGS data available from various cancer cell lines presented us with the opportunity to assess the performance of different methods using orthogonal information. We gathered 15 scRNA-seq datasets covering eight cancer cell lines with somatic mutations from WES available, and compared five previously published methods [10, 12, 15, 16] with varying strategies to benchmark against the RESA pipeline. Across all 15 datasets, both RESA and RESA-jLR consistently demonstrated substantially higher precision compared to other methods, achieving an average precision of 0.75 (Fig. 4a, Additional file 1: Fig. S12), while RESA-jLR increased sensitivity with slightly decreased precision compared to RESA alone (Fig. 4a, Additional file 1: Fig. S13). The high precision of RESA and RESA-jLR made the identified somatic mutations much more reliable for downstream analysis and interpretations, despite suboptimal sensitivity. The other methods showed variable sensitivity but dramatically lower precision, which diminished the value of these methods (Fig. 4a). Additionally, RESA and RESA-jLR outperformed other methods overall, with RESA-jLR showing marginally higher and more stable F0.5 performance (Fig. 4b). The best-performing method, other than RESA, is the method used by Maynard et al. [15] (Methods), which mainly focused on filtering against a curated somatic mutation whitelist combined with tissue source information. Although this filtering of mutations is intuitively reasonable, however, the performances of the Maynard et al. method varied dramatically depending on the dataset used, which confirmed the sporadic nature of cancer somatic mutations in a sample specific manner. In contrast, the somatic mutations identified by RESA between datasets of the same cell line showed remarkable consistency (Additional file 1: Fig. S14b).

Notably, RESA effectively identified somatic SNVs across cancer cells with varying mutation burdens despite sensitivity differences (Fig. 4a, Additional file 1: Fig. S14d). In particular, in the case of the JURKAT cell line, RESA detected a median of 449 and 387 somatic SNVs per cell in the SMART-seq and TARGET-seq datasets, respectively. These numbers were over 100 times higher than the somatic mutations detected in experimental multi-omic profiling (Additional file 1: Fig. S14c, Additional file 3: Table S1, Additional file 4: Table S1) [3, 4].

Next, we assessed the population-wise mutational spectrum captured by different methods against the WES mutational spectrum, and all expressed SNVs that can potentially be detected in the corresponding scRNA-seq data. We observed that expressed SNVs generally captured the exonic mutational spectra well, with lower numbers of SNVs detected and sparser presentation for low mutation burden samples (Fig. 4c, Additional file 1: Fig. S14d). Expressed SNVs detected by RESA-jLR reproduced the mutational spectrum of all expressed SNVs faithfully, particularly in samples with a high mutation burden (Fig. 4c, Additional file 1: Fig. S14d). In contrast, Maynard 2020 [15], the best-performing method in precision among all other methods, failed to capture the mutational spectra of either expressed or all exonic SNVs (Fig. 4c, Additional file 1: Fig. S14d). Therefore, expressed SNVs detected by RESA-jLR from the scRNA-seq data can be used to assess the genomic mutational spectra, particularly in samples with a high mutation burden.

### RESA achieved higher precision across tumor tissue datasets

**Fig. 5:**
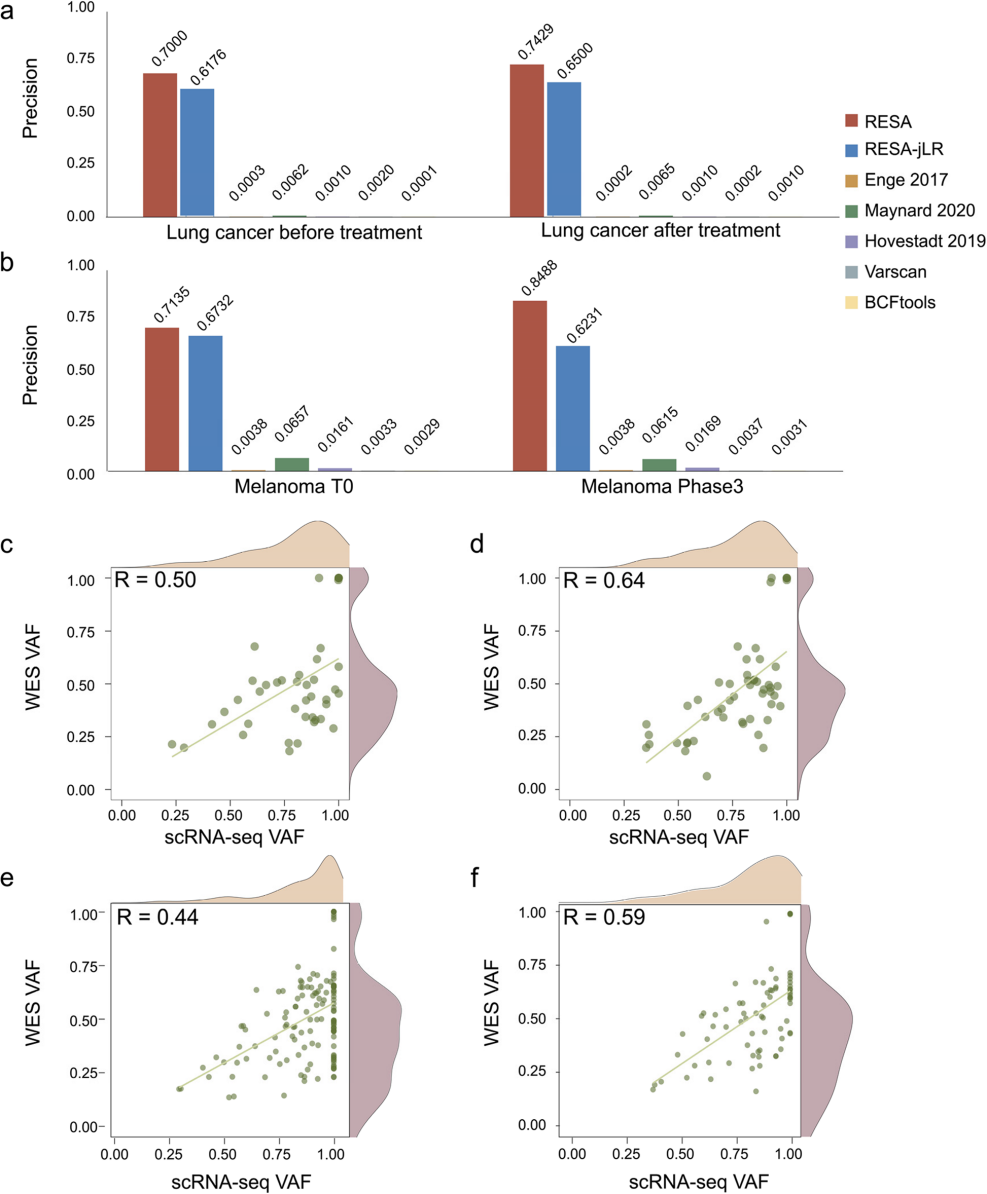
Benchmark RESA to other methods in PDX tumor datasets. **a** The bar plots illustrate precisions of RESA, RESA-jLR and other methods in a lung cancer PDX tumor from the same patient with 2 replicates. **b** The bar plots illustrate precisions of RESA, RESA-jLR and other methods in melanoma PDX datasets without treatment (T0) and after treatment (Phase3), **c**, **d**, **e**, **f**, Scatter plots showing VAF correlation of SNVs detected by RESA between scRNA-seq and WES in the lung cancer tumor sample with the replicate 1 (**c**) and replicate 2 (**d**), and melanoma datasets without treatment (**e**) and with treatment (**f**)

To further evaluate RESA's performance in real tumor data, we collected four scRNA-seq datasets with matching WES data from two independent studies involving different cancer types. These datasets include two replicates of lung adenocarcinoma tumor xenograft [25], and melanoma patient-derived xenograft (PDX) before and after RAF/MEK inhibitor treatment [36]. Again, RESA and RESA-jLR achieved the highest precision and F0.5 scores over other methods (Fig. 5a, b, Additional file 1: Fig. S15, Additional file 1: Fig. S16). In particular, in the lung cancer datasets with matched normal WES available, RESA was able to obtain higher performance using germline variants from matched normal than using publicly available SNP databases to filter out germline variants (Additional file 1: Fig. S17), highlighting that germline variants from matched normal should be applied in RESA whenever such data is available. Despite the likelihood of primary tumor samples being more heterogeneous than cancer cell lines, RESA maintained a positive correlation between scRNA-seq VAF and WES VAF, even with large variations in the WES VAF (Fig. 5c).

Melanoma tumors are characterized by UV signatures in their mutational spectra, which can serve as an additional indicator to assess the reliability of RESA. In the above-mentioned time-course study of RAF/MEK inhibition resistance using scRNA-seq datasets from the melanoma PDX model, over 600 single cells were profiled across four time points: T0 (tumor before treatment), phase 1 (tumor shrinking stage after treatment), phase 2 (minimal residual disease stage), and phase 3 (relapsed stage after drug treatment) [36]. RESA accurately reproduced the typical mutational spectrum of UV signature in all melanoma datasets (Additional file 1: Fig. S18a). Interestingly, the UV signature remains strong with little variation across different clusters or time points of the cell population, despite expression heterogeneity of somatic SNVs and involved genes (Additional file 1: Fig. S18c). This is consistent with previous knowledge that treatment with mechanism of action (MOA) not involving DNA replication and repair, e.g. BRAF inhibitor dabrafenib, is not expected to induce large mutational changes [36]. In addition, we identified the known BRAF V600E driver mutation present in the sample using RESA (Additional file 5: Table S1), further validating the reliability of RESA.

### RESA identifies drug resistance-associated genes with expressed somatic mutations in a melanoma PDX scRNA-seq dataset

**Fig. 6:**
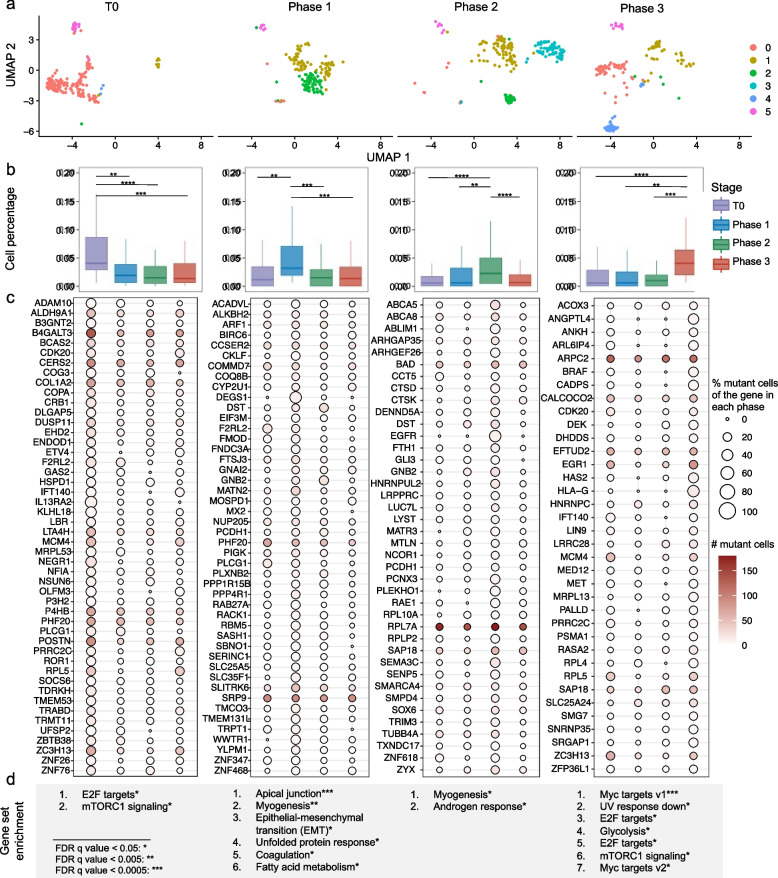
Somatic SNVs enriched in specific stages using RESA. **a** UMAP embedding of scRNA-seq profiles of each stage. **b** Distributions of percentages of cells harboring stage-specific mutations of each stage. (n.s.: *p* > 0.05, *: *p* <  = 0.05, **: *p* <  = 0.01, ***: *p* <  = 0.001, ****: *p* <  = 0.0001) **c** Aggregate expression of log_2_ of transcripts per 10,000 reads (color bar) for stage-specific genes detected in more than 10 cells and the number of cells harboring the mutation in each stage (cycle). **d** List of gene set enrichment results for each stage (MSigDB hallmark)

We applied RESA-jLR to the scRNA-seq datasets of the melanoma PDX model, where we identified 575 unique somatic SNVs and their corresponding 524 genes (Methods, Additional file 5: Table S1). To investigate the interplay between mutational heterogeneity and transcriptional heterogeneity, we further analyzed the dataset using graph-based clustering to summarize the gene expression heterogeneity (Additional file 1: Fig. S18b). By overlaying the four-time points onto the clusters, we identified both shared and unique clusters across timepoints (Fig. 6a), which confirmed the transcriptional heterogeneity in the scRNA-seq of the melanoma samples throughout different drug treatment stages. For example, cluster 3 represented unique cell groups at the MRD stage (phase 2), whereas cluster 2 showed expression patterns shared by both the tumor shrinking stage (phase 1) and MRD stage (phase 2). We observed that during the MRD stage of phase 2, cell clusters of 0,1,2,3,5 were all present, indicating a coexistence of heterogeneous subclones rather than the dominance of a single subclone despite minimal tumor size. Thus, our gene expression reanalysis validates the existence of multiple subclones with distinct gene expression signatures across different tumor stages.

In order to study the mutational heterogeneity during drug response, we defined "stage-specific" somatic mutations as follows (Fig. 6b,c). While most mutations were found present across all stages, we reasoned that subclonal mutations that contribute to different stages of the tumor should be non-uniformly present. Based on this assumption, we counted the number of cells carrying mutations in each tumor stage and calculated the enrichment accordingly. We then identified mutated genes that were enriched in specific tumor stages of the cell population compared to their wild-type counterparts. Specifically, we detected 72, 75, 88, and 63 mutated genes for the respective stages and focused our following analysis on mutated genes (Fig. 6b,c, Additional file 6: Table S1, Methods).

To investigate the mutated gene expression signatures, we tested GSEA curated cancer hallmarks enriched for the stage-specific mutated genes (Fig. 6d). As expected, mutated genes were enriched in pathways associated with melanoma development and progression, including the p53 pathway, epithelial to mesenchymal transition (EMT) pathway, mTORC1 signaling, androgen response pathway, and UV response [39–44]. Notably, when comparing hallmarks of mutated genes across different stages, the androgen response pathway, which is associated with melanoma tumor growth and invasion, was enriched exclusively in the MRD stage (Fig. 6d). Recent clinical research has reported that drug resistance is associated with sex, and males have shorter survival after BRAF-inhibitor monotherapy [45, 46]. Additionally, androgen receptor signaling is associated with the resistance of targeting BRAF [45]. Thus, the androgen response pathway may serve as a crucial mechanism for tumor survival in such cases. Therefore, cancer hallmarks that may be disturbed by expressed somatic SNVs may provide valuable orthogonal information beyond expression variation.

## Discussion

Identifying somatic mutations directly from scRNA-seq data has been a long-standing challenge. While attempts have been made, no computational method has been proven widely applicable. We report a computational framework named RESA, which identifies expressed somatic SNVs with high precision directly from the scRNA-seq data. RESA can effectively filter out noise and artefacts generated during the experimental procedure, thereby achieving high precision. In addition, RESA applies a joint logistic regression to expand the putative somatic mutations, which helps increase detection sensitivity while maintaining high precision. We comprehensively benchmarked RESA across datasets both in vitro and in vivo, and demonstrated the reliability of RESA in different scenarios.

In addition to evaluating individual SNVs, RESA also attempts to assess the mutational spectrum of expressed SNVs. High precision detection of somatic SNVs enables reliable mutational signature analysis of the expressed SNVs in scRNA-seq data, thus providing insights into the mechanistic biological processes involved in cancer progression, or revealing potential therapeutic opportunities in the sample of interest.

Balancing precision and sensitivity is tricky. Precision measures the proportion of true somatic mutations identified by RESA out of all somatic mutations suggested by RESA (Additional file 1: Fig. S1). On the other hand, sensitivity measures the proportion of true somatic mutations out of all somatic mutations carried in scRNA-seq data. Previous methods mostly achieve high sensitivity, but their low precisions lead to an excessive amount of noise and artefacts (Additional file 1: Fig. S1). The main purpose of our model is to identify higly reliable somatic mutation, which emphasizes more on precision over sensitivity. So that we can be very confident in the RESA-suggested somatic mutations in cases without matched WES/WGS for evaluation and improved the reliability of downstream analysis. To compensate for the loss of sensitivity, and increase sensitivity, we developed the RESA-jLR modeling step. In addition, we used the F0.5 score to reconcile the performance evaluation balance between precision and sensitivity. As demonstrated in our results, RESA-jLR achieves better performances than RESA in cases of high mutation burden or high sequencing coverage per cell.

VAF represents the fraction of alleles containing mutations. In WES, the VAF value is affected by tumor purity and clonality. In bRNA-seq, the VAF interpretation is further complicated due to allelic and stochastic expression and the abundance of genes containing mutations. However, the VAF estimate from scRNA-seq might be perceived as the deconvolution of bRNA for VAF without the complication of tumor purity as long as only cancer cells are assessed. Interestingly, we observed a positive correlation between scRNA-seq VAF and VAF from both bRNA-seq (Fig. 2d) and WES (Fig. 2e) in the A375 cell line data. These results suggest that scRNA-seq VAF might be indicative of the mutant VAF at the bulk level. Notably, we detected somatic mutations from scRNA-seq with a WES VAF as low as 0.13 (Additional file 1: Fig. S3).

Detection of expressed SNVs using RESA may suffer from several limitations. RESA works best in scRNA-seq data that are sequenced relatively deep (1–3 million reads per cell in general), or in samples with relatively high mutation burden. Application to other widely used scRNA-seq technologies like 10X genomics and drop-seq might thus be limited by the nature of shallow sequencing and biased gene region coverage. However, recent studies have made efforts to address the coverage issue by combining bam files of the same cell type in 10X Genomics datasets to detect somatic mutations [47]. Taking this into consideration, we acknowledge that RESA could be improved by incorporating this strategy in the future, thereby expanding its potential application areas. Another potential limitation is that this approach is designed to detect clonal somatic mutations instead of rare mutations in the population. Empirically we found that WES VAF above 0.1 can be detectable in scRNA-seq data and RESA. In addition, due to the nature of the scRNA-seq experiment, only mutations with relatively high expression are potentially detectable. Lastly, some somatic mutations expressed in a single cell might not be detectable through WES/WGS, leading to a decreased precision value that underestimates the real precision. Moreover, the clonal divergence between cell cultures that generated WES/WGS data vs. scRNA-seq data might also decrease the precision. Thus, the limited sensitivity of scRNA-seq data as well as RESA might potentially limit its application.

The success of RESA in high precision somatic mutation detection from scRNA-seq data highlights the critical importance of cellular recurrence, whereas prioritizing based on functional prediction or stratification from curated whitelist mutations does not always work well. This is the first study, to the best of our knowledge, that made a direct comparison among the methods emphasizing the above aspects and delivering distinctive results. As more datasets, especially datasets with both scRNA-seq and WES/WEG profiling on the same sample, become available, new methods may be developed with better performance. We believe RESA will provide valuable information to facilitate single cell level genotype to phenotype study in the future.

## Conclusions

In summary, we introduce a computational framework package named RESA that identifies expressed somatic mutations from scRNA-seq de novo. RESA effectively filters out noise and artefacts identified through common variant calling pipelines. We demonstrated the remarkable improvement in precision and F0.5 score of RESA against other methods across multiple test case scenarios. We showcased the application of RESA-jLR to provide novel insights into the potential mutational mechanisms underlying melanoma MRD, and we believe RESA is highly valuable to providing orthogonal insights into the intratumor heterogeneity studies in scRNA-seq datasets.

### Supplementary Information

**Additional file 1: **Includes 18 figures of additional results. The names of the figure are: **Figure S1. **Workflow illustrates the different strategies between RESA and other methods; **Figure S2.** The correlation between site-specific depth and number of detectable SNVs in scRNA-seq; **Figure S3. **Scatter plots showing VAF correlation of SNVs detected by RESA between scRNA-seq and WES in the A375 cell line; **Figure S4.** Full length scRNA-seq sequencing procedure, steps where noise and artefacts may be introduced are highlighted; **Figure S5. **Comparison between identified somatic SNVs and identified noise and artefacts; **Figure S6.** Scatter plots showing VAF correlation of SNVs detected by RESA between scRNA-seq and WES, as well as scRNA-seq and bRNA-seq in the A375 cell line and LNCaP cell line; **Figure S7.** AUC values of the joint logistic regression model prediction on the test set in 15 tested datasets;** Figure S8.** Correlation of feature weights in quality-based and sequence-based logistic regression; **Figure S9. **The distribution of million reads per cell in in silico spike-in datasets of 1-month-old, 5-year-old, 6-year-old donors; **Figure S10. **The bar plot illustrates the percentage change of the number of expressed SNVs detected by RESA-jLR against RESA-identified SNVs in the in silico spike-in scRNA-seq dataset of a 5-year-old child; **Figure S11.** The bar plot illustrates sensitivity in the in silico spike-in scRNA-seq dataset of 1-month-old, 5-year-old, 6-year-old donors; **Figure S12.** Boxplots showing precisions (top) and sensitivities (bottom) of different methods in identifying positive somatic SNVs using WES data as ground truth across 15 scRNA-seq datasets; **Figure S13. **The bar plot illustrates the sensitivity of 15 cell line datasets;** Figure S14. **Evaluating RESA performance with comparison to other methods; **Figure S15.** The bar plot illustrates sensitivity in PDX tumor datasets; **Figure S16.** Bar plots illustrate the F0.5 scores of RESA, RESA-jLR, and other methods in PDX tumor datasets; **Figure S17.** Lung cancer PDX tumor datasets with the matched normal in two groups of independent cells; **Figure S18. **Reanalysis of a melanoma scRNA-seq dataset using RESA.

**Additional file 2:** Includes 1 table for cell line data sets used in the study. The name of the table is:** Table S1. **Somatic SNVs detecting by RESA to full-length scRNA-seq data from 15 datasets.

**Additional file 3:** Includes 1 table of the annotation for cell line data sets used in the study. The name of the table is:** Table S1. **Annotation of each mutation in 15 cell line datasets detected by RESA.

**Additional file 4:** Includes 1 table of the statistical summary for cell line data sets. The name of the table is:** Table S1.** Summary statistic table of mutations detected by RESA.

**Additional file 5:** Includes 1 table for melanoma data sets. The name of the table is:** Table S1.** 575 unique somatic SNVs of scRNA-seq of the melanoma detected by RESA, which mapped to 524 genes.

**Additional file 6:** Includes 1 table of the stage enrichment for melanoma data sets. The name of the table is:** Table S1.** P values of the Hypergeometric test for stage-specific somatic SNVs enrichment in each stage.

## Data Availability

The WES data and bulk RNA-seq data for cancer cell line LNCaP were downloaded through Genomic Data Commons (https://gdc.cancer.gov/). The exonic somatic mutations for all cancer cell lines were downloaded from CCLE_20Q1_mutations [20]. The scRNA-seq for cancer cell lines were downloaded from GSE105451 [4], GSE99795 [21], GSE76312 [5], GSE150993 [22], GSE108383 [23], GSE140440 [24], and GSE69405 [25] respectively. The lung cancer scRNA-seq data and WES were downloaded from GSE69405 [25]. The human tissue scRNA-seq data of melanoma were downloaded from GSE81547 [35]. The matched WES of melanoma PDX was downloaded from EGAD00001007749 [36]. The source code for the RESA pipeline is available at https://github.com/ShenLab-Genomics/RESA [26].

## Abbreviations

scRNA-seq: Single-cell RNA sequencing
RESA: Recurrently Expressed SNV Analysis
MRD: Minimal residual disease
WGS: Whole genome sequencing
WES: Whole exome sequencing
RESA-jLR: RESA followed by a joint logistic regression
VAF: Variant allele frequency
bRNA-seq: Bulk RNA-seq
PDX: Patient-derived xenograft
MOA: Mechanism of action
EMT: Epithelial to mesenchymal transition
scWGS: Single cell whole genome sequencing
LUAD: Lung adenocarcinoma
