# Supplementary material for: De novo identification of expressed cancer somatic mutations from single-cell RNA sequencing data
Source: Genome Med. 2023 Dec 18;15:115. doi: 10.1186/s13073-023-01269-1 (PMC10726641; doi:10.1186/s13073-023-01269-1)
Supplement: Supplementary file 1 — Additional file 1: Includes 18 figures of additional results. The names of the figure are: Figure S1. Workflow illustrates the different strategies between RESA and other methods; Figure S2. The correlation between site-specific depth and number of detectable SNVs in scRNA-seq; Figure S3. Scatter plots showing VAF correlation of SNVs detected by RESA between scRNA-seq and WES in the A375 cell line; Figure S4. Full length scRNA-seq sequencing procedure, steps where noise and artefacts may be introduced are highlighted; Figure S5. Comparison between identified somatic SNVs and identified noise and artefacts; Figure S6. Scatter plots showing VAF correlation of SNVs detected by RESA between scRNA-seq and WES, as well as scRNA-seq and bRNA-seq in the A375 cell line and LNCaP cell line; Figure S7. AUC values of the joint logistic regression model prediction on the test set in 15 tested datasets; Figure S8. Correlation of feature weights in quality-based and sequence-based logistic regression; Figure S9. The distribution of million reads per cell in in silico spike-in datasets of 1-month-old, 5-year-old, 6-year-old donors; Figure S10. The bar plot illustrates the percentage change of the number of expressed SNVs detected by RESA-jLR against RESA-identified SNVs in the in silico spike-in scRNA-seq dataset of a 5-year-old child; Figure S11. The bar plot illustrates sensitivity in the in silico spike-in scRNA-seq dataset of 1-month-old, 5-year-old, 6-year-old donors; Figure S12. Boxplots showing precisions (top) and sensitivities (bottom) of different methods in identifying positive somatic SNVs using WES data as ground truth across 15 scRNA-seq datasets; Figure S13. The bar plot illustrates the sensitivity of 15 cell line datasets; Figure S14. Evaluating RESA performance with comparison to other methods; Figure S15. The bar plot illustrates sensitivity in PDX tumor datasets; Figure S16. Bar plots illustrate the F0.5 scores of RESA, RESA-jLR, and other methods in PDX tumor da [file 13073_2023_1269_MOESM1_ESM.pdf]

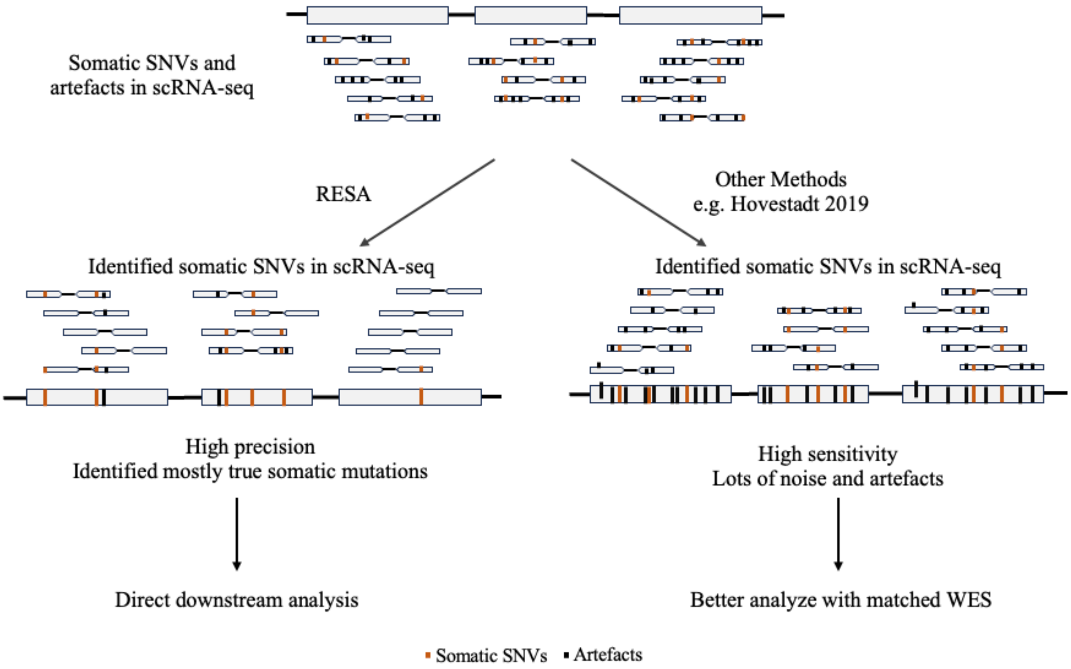

Figure S1. Workflow illustrates the different strategies between RESA and other methods.

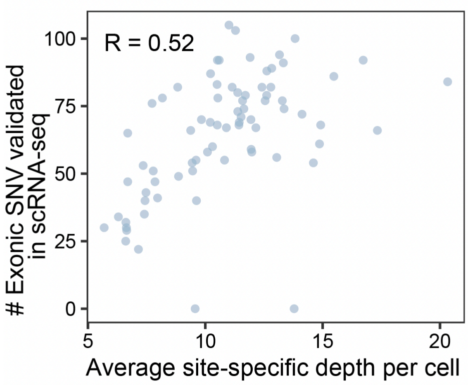

Figure S2. The correlation between site-specific depth and number of detectable SNVs in scRNA-seq.

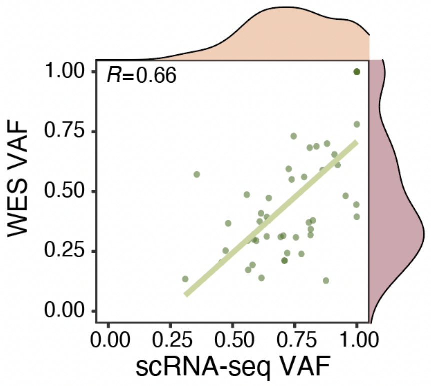

Figure S3. Scatter plots showing VAF correlation of SNVs detected by RESA between scRNA-seq and WES in the A375 cell line.

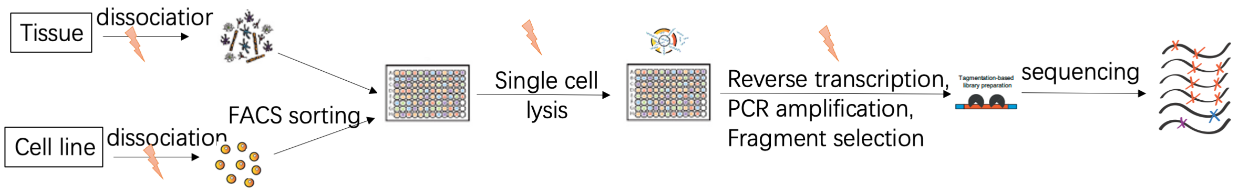

Figure S4. Full length scRNA-seq sequencing procedure, steps where noise and artefacts may be introduced are highlighted.

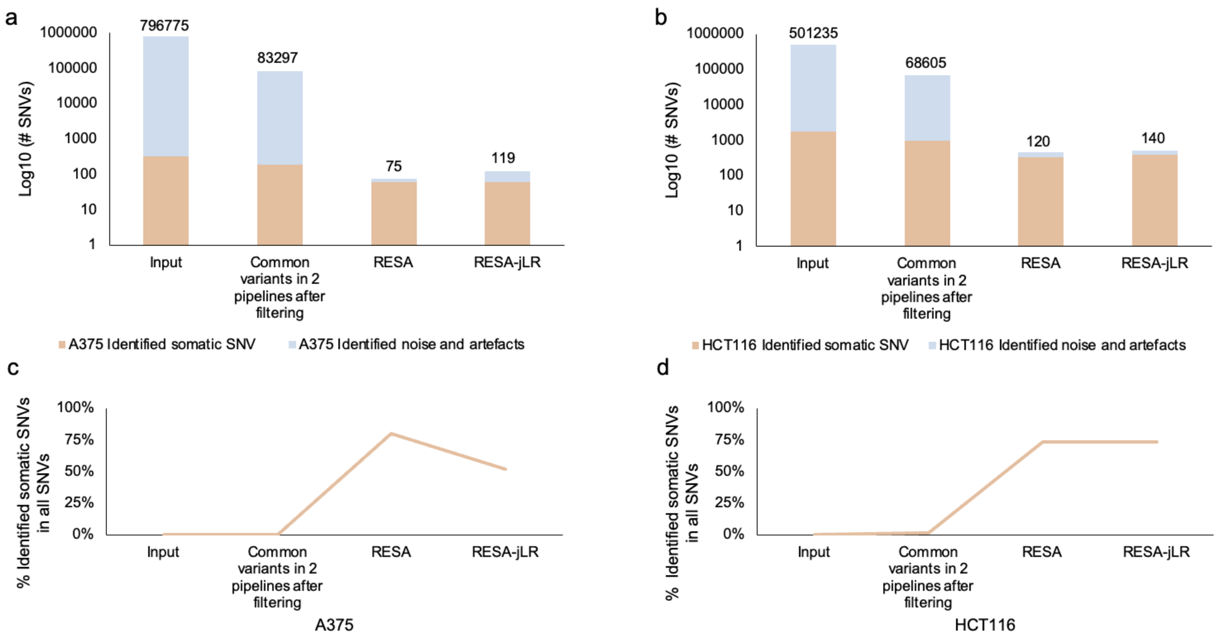

Figure S5. Comparison between identified somatic SNVs and identified noise and artefacts. a, b, Bar plots showing the number of identified somatic SNVs and identified noise and artefacts changed after different filtering conditions in the A375 cell line (a) and HCT116

cell line (b). c, d, Line charts illustrating the percentage of identified somatic SNVs in all SNVs in the A375 cell line (c) and HCT116 cell line (d).

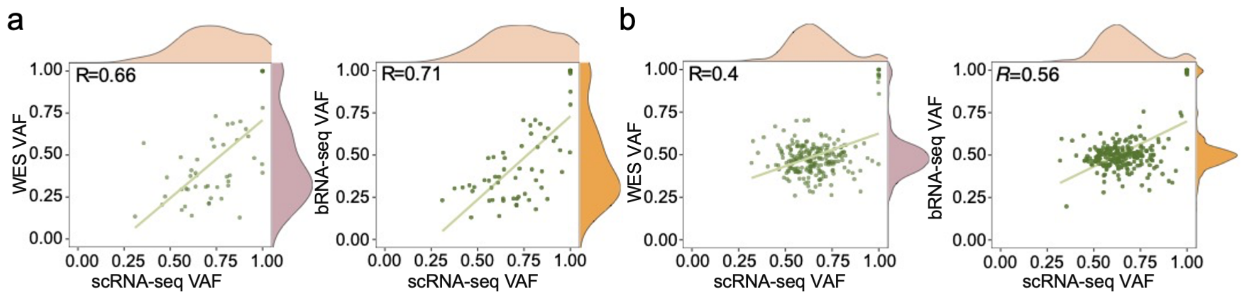

Figure S6. Scatter plots showing VAF correlation of SNVs detected by RESA between scRNA-seq and WES, as well as scRNA-seq and bRNA-seq in the A375 cell line (a) and LNCaP cell line (b).

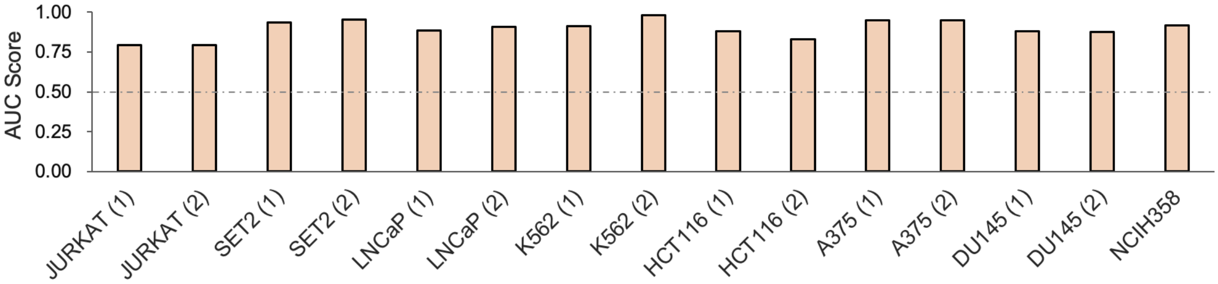

Figure S7. AUC values of the joint logistic regression model prediction on the test set in 15 tested datasets. Dashed line labeling at an AUC of 0.5 indicates the model is not able to distinguish between positive somatic SNVs and artefacts, as its predictive performance is no better than making random predictions.

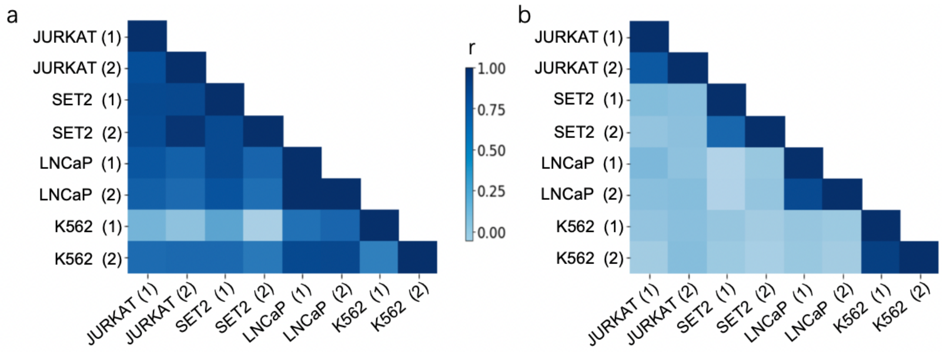

Figure S8. Correlation of feature weights in quality-based and sequence-based logistic regression. a, High correlation of feature weights across most datasets observed in quality-

based logistic regression. b, Cell type specific correlation of feature weights observed in sequence-based logistic regression.

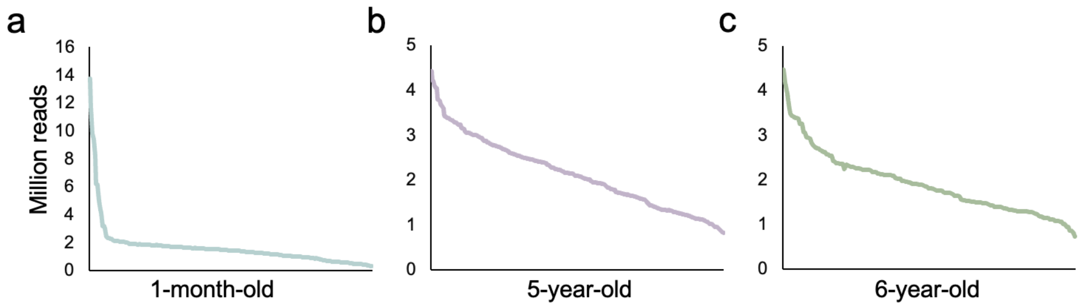

Figure S9. The distribution of million reads per cell in *in silico* spike-in datasets of 1-month-old (a), 5-year-old (b), 6-year-old(c) donors.

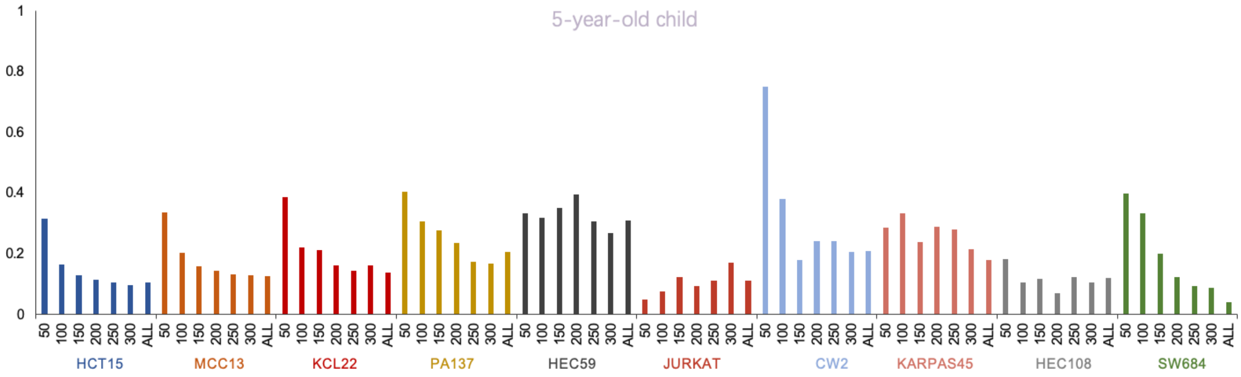

Figure S10. The bar plot illustrates the percentage change of the number of expressed SNVs detected by RESA-jLR against RESA identified SNVs in the *in silico* spike-in scRNA-seq dataset of a 5-year-old child.

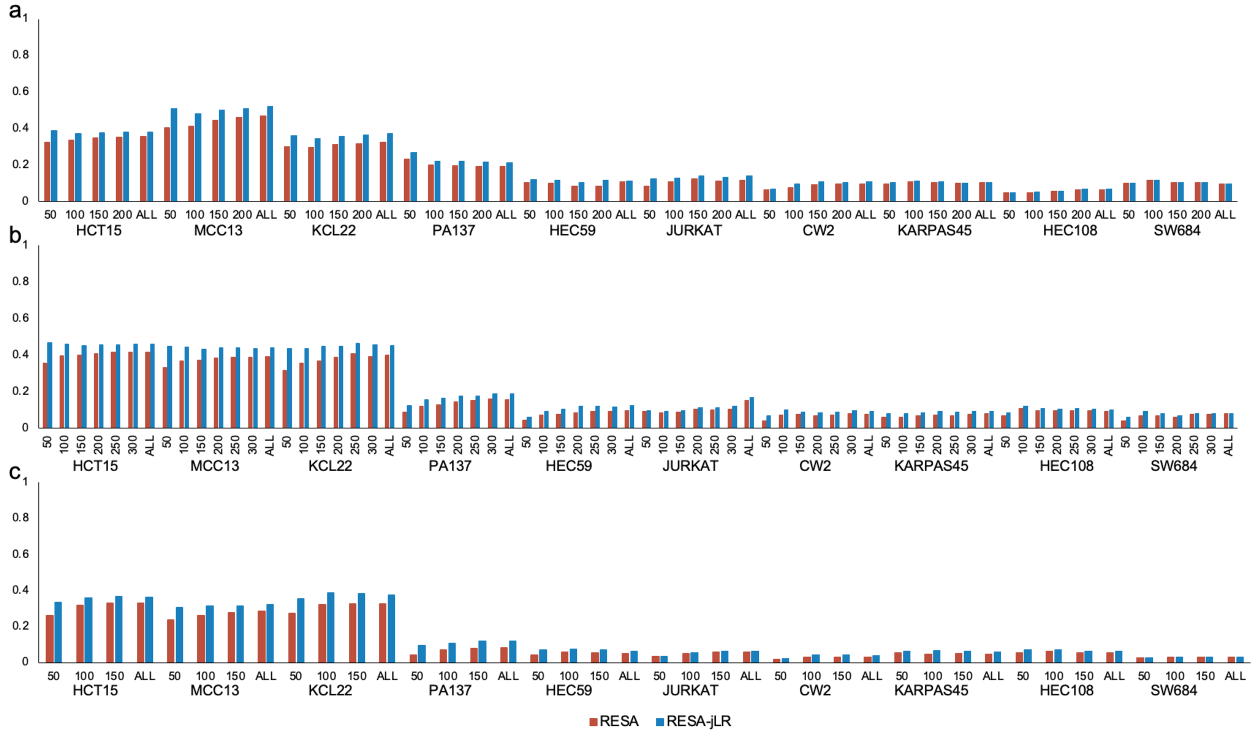

Figure S11. The bar plot illustrates sensitivity in the *in silico* spike-in scRNA-seq dataset of 1-month-old (a), 5-year-old (b), 6-year-old(c) donors.

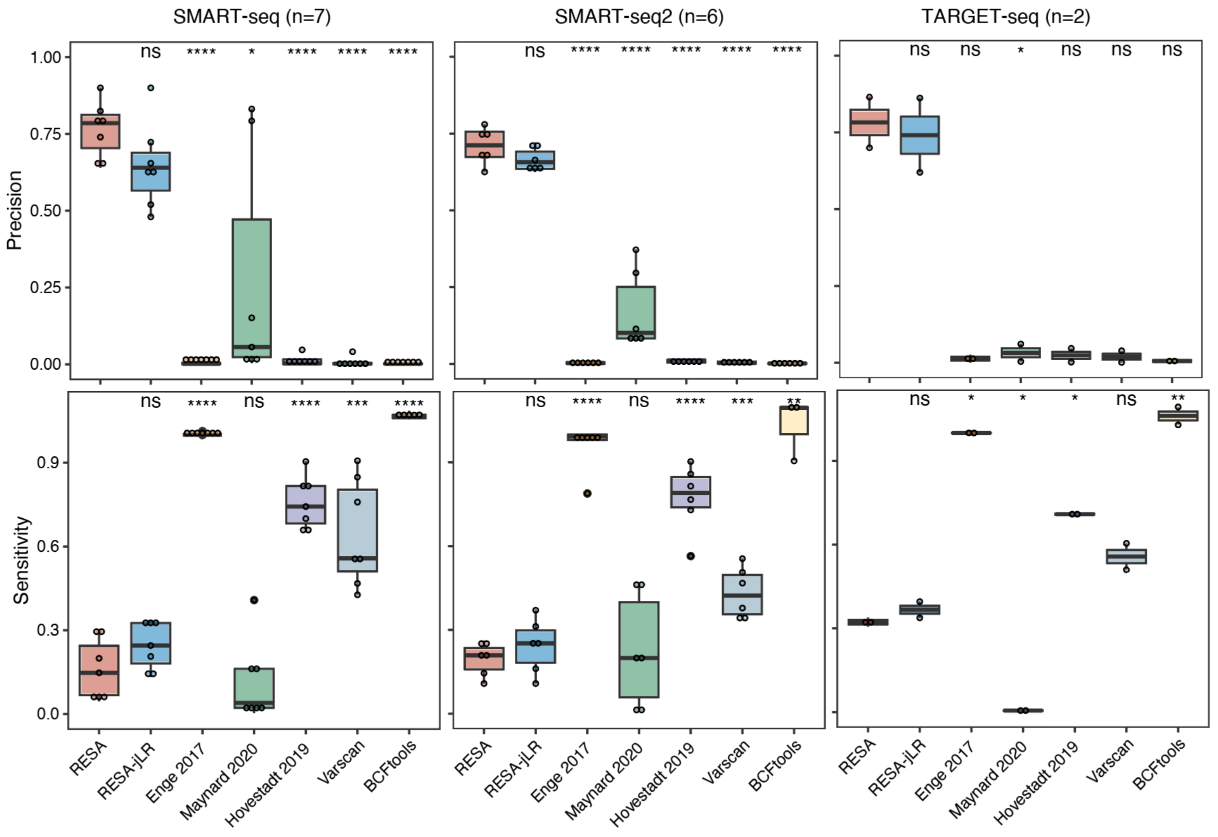

Figure S12. Boxplots showing precisions (top) and sensitivities (bottom) of different methods in identifying positive somatic SNVs using WES data as ground truth across 15

scRNA-seq datasets. Each of the grouping variables is compared to the RESA group using the student t-test and statistical significances are shown above each group. (n.s.: p>0.05, *: p <= 0.05, **: p <= 0.01, ***: p <= 0.001, ****: p <= 0.0001)

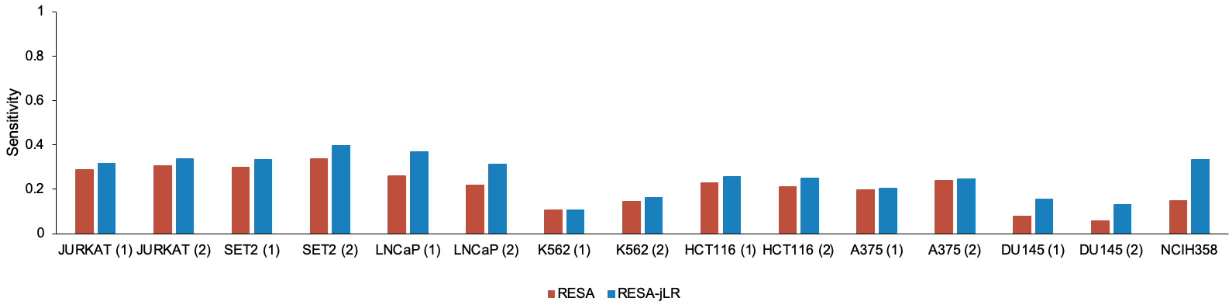

Figure S13. The bar plot illustrates the sensitivity of 15 cell line datasets.

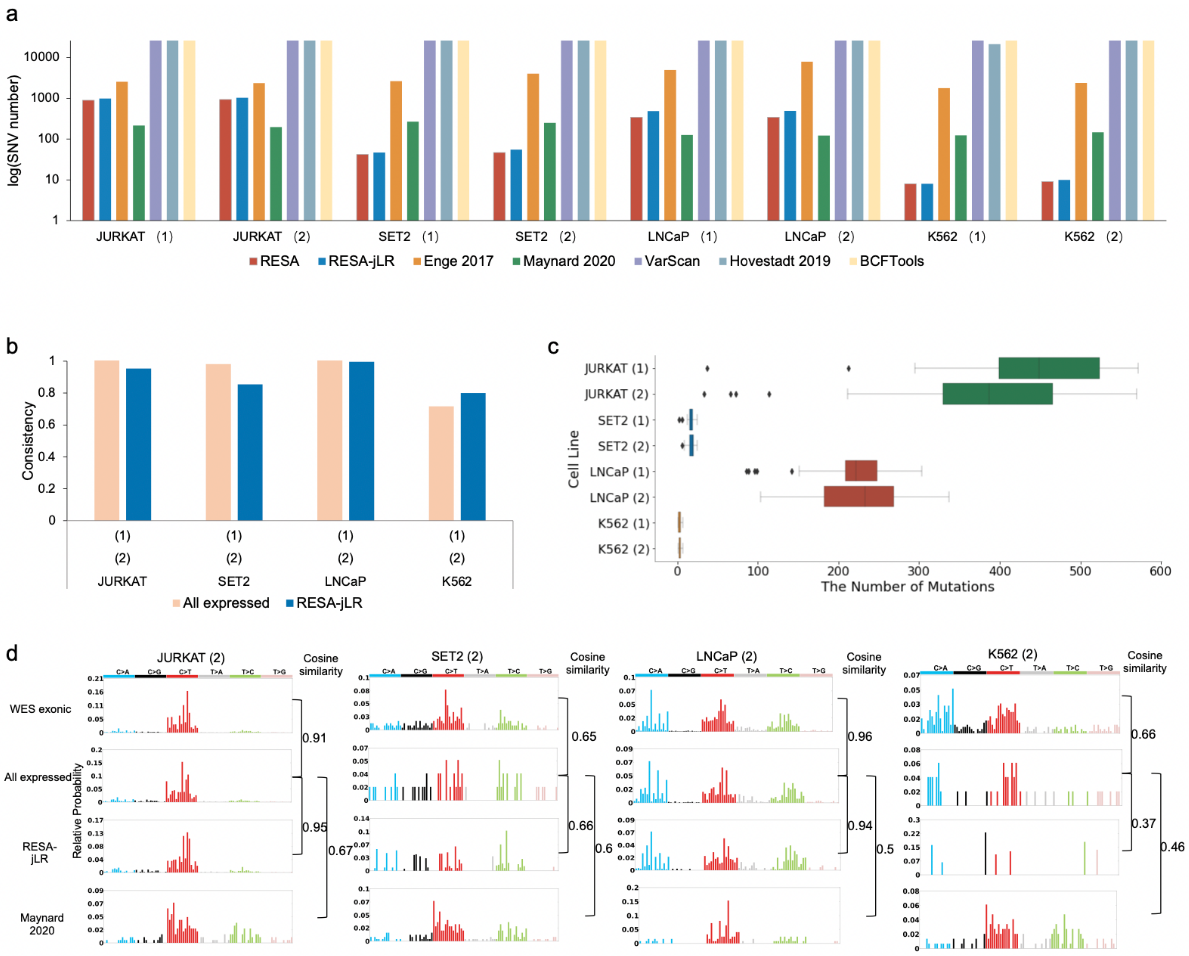

Figure S14. Evaluating RESA performance with comparison to other methods. a. The bar plot showing the total number of mutations (in log10 format) detected by different methods across all 8 datasets. b. The bar plot showing the consistency across 4 cell lines. c. Boxplot showing the number of mutations per cell as detected by RESA-jLR across all 8

datasets. d. Mutation spectra of somatic SNVs identified using WES exonic, all expressed SNVs, RESA-jLR, and Maynard 2020 approach across 4 datasets. Pairwised cosine similarity score was shown next to the brackets.

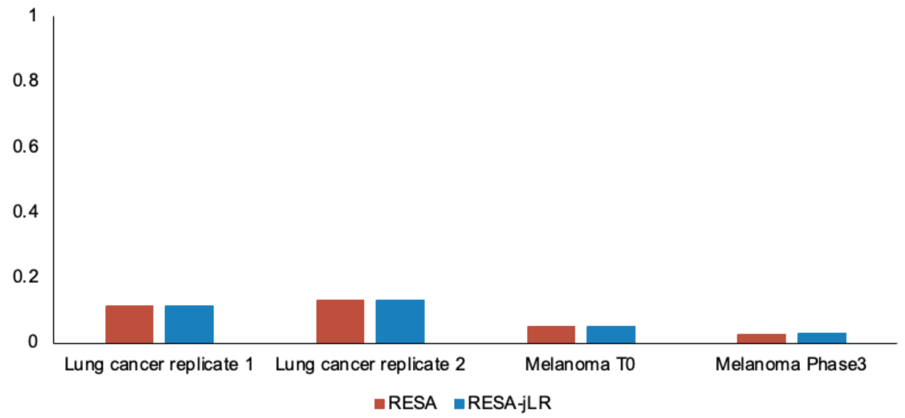

Figure S15. The bar plot illustrates sensitivity in PDX tumor datasets.

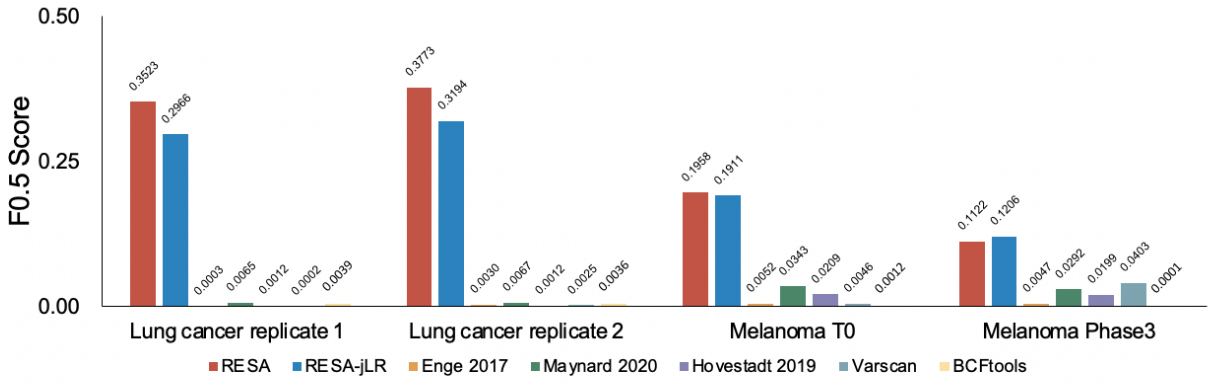

Figure S16. Bar plots illustrate the F0.5 scores of RESA, RESA-jLR, and other methods in PDX tumor datasets.

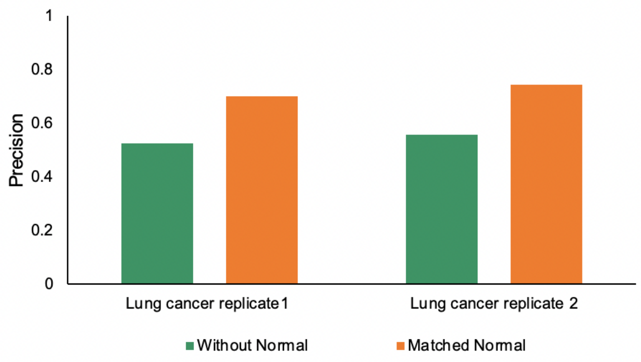

Figure S17. Lung cancer PDX tumor datasets with the matched normal in two groups of independent cells.

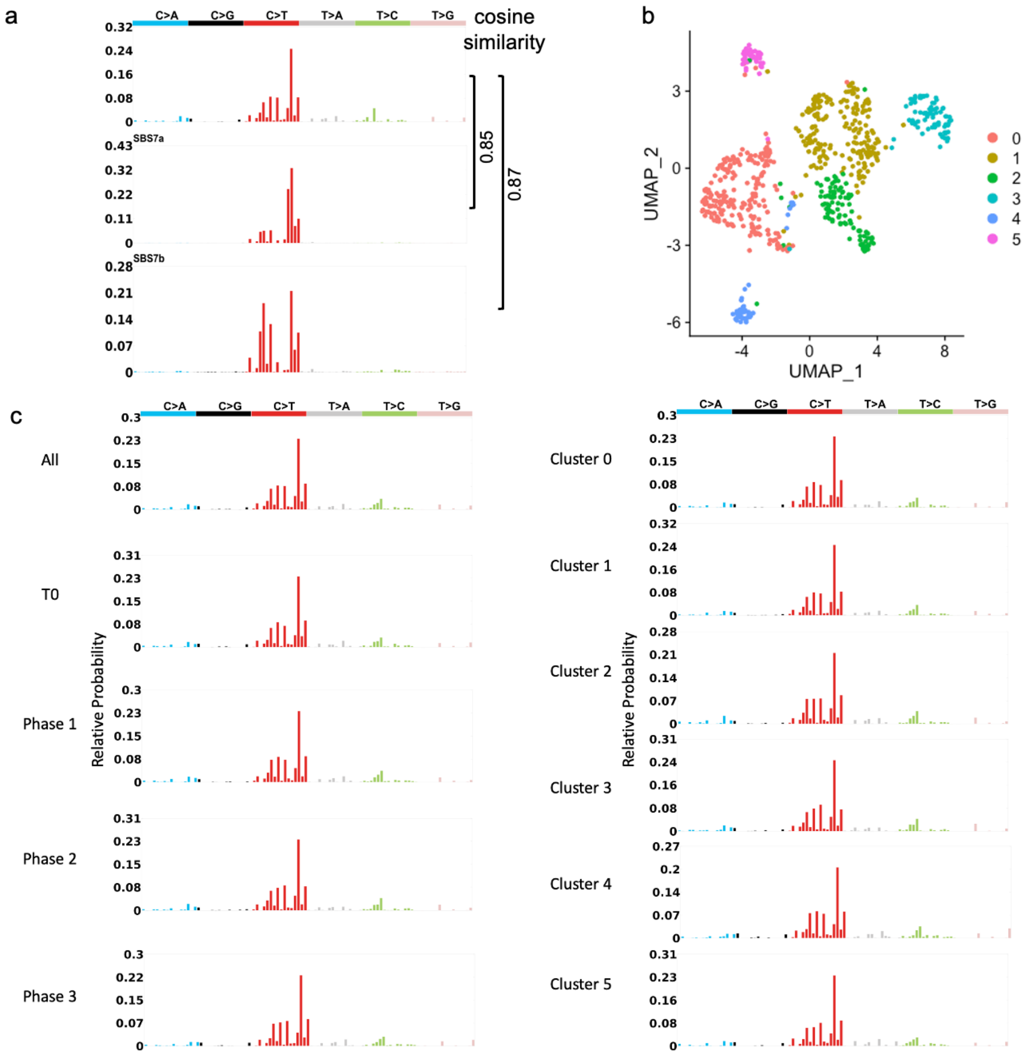

Figure S18. Reanalysis of a melanoma scRNA-seq dataset using RESA. a. Mutational spectra of somatic variants identified by RESA-jLR recapitulate UV-dominated signature. Cosine similarity to COSMIC cancer mutation signatures 7a and 7b is labeled on the right. b. UMAP embedding 6 expression clusters. c. Mutation spectra of somatic SNVs identified by RESA for cells in different tumor stages and different expression clusters.
